# Biopsychosocial Contributors to Parent Behaviors during Child Venipuncture

**DOI:** 10.3390/children9071000

**Published:** 2022-07-02

**Authors:** Kaytlin L. Constantin, Rachel L. Moline, Rebecca Pillai Riddell, Jeffrey R. Spence, C. Meghan McMurtry

**Affiliations:** 1 Department of Psychology, University of Guelph, Guelph, ON N1G 2W1, Canada; rmoline@uoguelph.ca (R.L.M.); spencejr@uoguelph.ca (J.R.S.); cmcmurtr@uoguelph.ca (C.M.M.); 2 Department of Psychology, York University, Toronto, ON M3J 1P3, Canada; rpr@yorku.ca; 3 Department of Psychiatry Research, The Hospital for Sick Children, Toronto, ON M5G 1X8, Canada; 4 Department of Psychiatry, University of Toronto, Toronto, ON M5T 1R8, Canada; 5 Pediatric Chronic Pain Program, McMaster Children’s Hospital, Hamilton, ON L8N 3Z5, Canada; 6 Department of Anesthesia, McMaster University, Hamilton, ON L8S 3L8, Canada; 7 Department of Paediatrics, Schulich School of Medicine & Dentistry, Western University, London, ON N6A 5W9, Canada

**Keywords:** acute pediatric pain, biopsychosocial, heart rate variability, verbal behaviors

## Abstract

Children’s needle-related distress is strongly related to parental verbal behaviors. Yet, empirical data supporting theorized contributors to parent behaviors in this context remain limited. This is the first study to collectively measure biological (heart rate variability; HRV), psychological (catastrophizing, anxiety), and social (child behaviors) contributors to parent verbal behaviors throughout pediatric venipuncture. HRV was used as a measure of emotion regulation capacity and examined as a moderator in the associations between parent psychological factors and their behaviors, and between child and parent behaviors. Sixty-one children aged 7 to 12 years who presented at an outpatient blood lab for venipuncture and a parent participated. Parent baseline HRV, state catastrophizing, and anxiety were measured prior to venipuncture. The procedure was video-recorded for later coding of pairs’ verbal behaviors. Strong associations emerged between child behaviors and parent behaviors. Baseline HRV moderated the association between parent catastrophizing and behavior. Social factors remain a strong influence related to parent behaviors. Psychologically, parent negative cognitions differentially related to parent behaviors based on their emotion regulation capacity. Biologically, low baseline HRV may increase the risk that certain parents engage in a constellation of behaviors that simultaneously direct their child’s attention toward the procedure and inadvertently communicate parental worry, fear, or concern.

## 1. Introduction

Rooted in a biopsychosocial framework, the affective-motivational model (AMM) of interpersonal pain dynamics highlights the interpersonal nature of pain [1]. Parents teach their children how to anticipate danger, the meaning of pain, and proactive responses [2]. Intentional self-management of pain develops slowly across childhood; children typically require prompting by adults to engage in coping behaviors, and without explicit guidance, children are more likely to experience distress from painful procedures [3]. Up to 64% of the variance in child procedure-related pain experiences relates to parent behaviors [4,5]. One constellation of parent procedural verbalizations tends to correlate with child coping (termed “coping-promoting”; i.e., humor, non-procedure related talk, and commands to engage in coping), while another tends to relate to greater child distress (“distress-promoting”; i.e., reassurance, empathy, apologies, criticism, and giving child control [5,6]). (***Note.*** For simplicity, we use the terms “distress-promoting” and “coping-promoting”, which are the terms consistent with the coding system used (i.e., CAMPIS, [3]); however, we acknowledge that this language implies directionality between parent and child behaviors that is not fully supported by existing data). Understanding of which parents are more likely to use behaviors related to child coping or distress remains limited [7].

The AMM proposes that the ways parents respond to their child’s displays of pain are shaped by their pain-related emotions, with emotion regulation central to facilitating behaviors sensitive to their child’s needs (other-oriented emotional responses, e.g., sympathy) versus their own (self-oriented responses, e.g., distress [1]). The model contends that these emotional responses, arising from cognitive appraisals (e.g., catastrophizing) and resulting in subjective affective states (e.g., anxiety), shape caregiving behaviors. Although parent anxiety and catastrophizing often result in unhelpful parent behaviors (e.g., [8,9,10]), emotion regulation may help explain inconsistent findings [11,12,13]. In particular, the model predicts that the extent to which parent behaviors match their child’s needs is contingent on the parent’s ability to shift flexibly between self- and other-oriented states; emotion regulation is central to this process. Heart rate variability (HRV; variation in time between inter-beat intervals) is commonly used to assess emotion regulation capacity [14]. High baseline HRV is indicative of greater flexibility in emotional responding [15,16]. Existing research on parental baseline HRV in the context of child acute pain are laboratory-based studies demonstrating HRV informs parent behaviors [17,18,19] (see [20,21] for parent–child HRV in a clinical context).

This study sought to explore biopsychosocial contributors to parent behaviors, including parent baseline HRV, cognitive-affective states, and child distress behaviors. We examined: (1) whether parent baseline HRV interacts with their cognitive-affective states to shape verbal behaviors; and (2) if parent baseline HRV is a moderator between child and parent behaviors. Guided by the AMM, we hypothesized: (i) parent anxiety and catastrophizing would show the strongest association to parent distress-promoting behaviors in parents with low (vs. high) baseline HRV; (ii) child distress behavior would demonstrate the strongest relation to parent distress-promoting behavior in parents with low baseline HRV. Parent coping-promoting behavior was examined in an exploratory way.

## 2. Materials and Methods

### 2.1. Participants

Two research ethics boards approved this study. Children (7 to 12 years) presenting at the outpatient blood lab at McMaster Children’s Hospital with a requisition form to complete a venipuncture and a parent were recruited. Inclusion criteria were: English proficiency in parent and child sufficient to read and answer questions, child undergoing venipuncture, and parents being a primary caregiver and who was accompanying their child during the venipuncture.

### 2.2. Procedure

This study falls under a single-site, two-arm, parallel-group RCT (#NCT03941717) and data collected as part of this larger study are presented in three other manuscripts examining: (1) how a brief mindfulness intervention affected child and parent venipuncture experiences [22]; (2) whether parent and child HRV differed as a function of venipuncture phase and by RCT group [20] and (3) biopsychosocial aspects of children’s venipuncture experience [23]. This paper explores a unique set of research aims related to parent baseline HRV, self-reported states, and child behavior to understand parent behaviors. The possible effects of the RCT were not the focus of this study; RCT group was included as a potential control variable (see the analytic plan for details related to how the RCT group was addressed in the analyses).

Families were greeted by two research assistants if they presented to the blood lab at McMaster Children’s Hospital with a child who seemed to be 7 years of age or older. Interested and eligible parents and children were given an overview of the study, after which parents provided written consent and children provided assent. The wireless electrocardiogram (ECG) equipment was placed on parents. Parents self-reported on state pain catastrophizing and anxiety through online survey software on a tablet. Parents and their child were randomly assigned to either a mindfulness group (i.e., brief mindfulness intervention; *n* = 31) or control group (i.e., unfocused attention task; *n* = 30), in which dyads listened to a 5-min audio recording (see [24] for protocol). Baseline HRV was acquired during the first 30 s of the audio. Next, parents joined their child for the procedure, which was provided as usual, apart from the procedure being videotaped, and parents wore a clip-on microphone and wireless ECG equipment during the procedure. As part of the larger study, children also wore wireless ECG equipment. Following data collection, transcripts of the pairs’ interactions were created for coding of verbal behavior (details below).

### 2.3. Measures

#### 2.3.1. Demographics

Parents provided information on their own and their child’s demographics. Parents were also asked to self-report on their caffeine, nicotine, and medication use as these can influence baseline HRV [25].

#### 2.3.2. Child–Adult Medical Procedure Interaction Scale (CAMPIS) and CAMPIS-R

The CAMPIS is an observational scale used to assess adult and child verbal behaviors during painful procedures [26]. The original CAMPIS consists of 35 codes, which were combined into 6 categories for the CAMPIS-R [6]. In the CAMPIS-R, child behaviors include: distress (e.g., crying, screaming), coping (e.g., nonprocedural talk, humor), and neutral (e.g., general condition related talk). Adult verbal behaviors (parent and healthcare provider) include: distress-promoting (e.g., reassurance, apologies), coping-promoting (e.g., nonprocedural talk, humor), and neutral (e.g., checking child’s status). This measure is considered well-established to capture adult–child interactions, with direct clinical utility and shows acceptable to excellent psychometric properties [6,27]. The pairs’ verbalizations during venipuncture were transcribed verbatim and used for coding.

##### Transcription

Based on video recordings, the primary transcriber transcribed utterances from the moment the child sat in the procedure chair for the venipuncture until they left the chair. A secondary transcriber reviewed the transcripts and recording for accuracy. Disagreements were resolved by a third transcriber. The venipuncture time ranged from 1.38 to 18.53 min (*M* = 6.10, *SD* = 3.27), which is the timeframe in which the verbalizations were transcribed and coded using the CAMPIS.

##### CAMPIS Training

Two coders were trained to identify all 35 adult and child verbal behaviors listed in the CAMPIS using didactic methods, including reviewing definitions and examples for each code. Once coders became familiar with all 35 of the verbal behavior codes, practice coding using sample transcripts from another data set occurred until 85% interrater reliability was reached.

##### CAMPIS Coding

The primary CAMPIS coder grouped together content according to thought units given the number of simultaneous and overlapping vocalizations. If a vocalization could not be identified, it was coded as “other” [26]. Consistent with CAMPIS coding instructions, when the same code could be used for successive vocalizations, it was only scored once unless interrupted by another speaker or code. Consistent with past work (e.g., [7,28]), coders first read through the entire transcript and coded obvious verbalizations, which was followed by watching the video to code the rest of the verbalizations.

Continuous event coding was applied to capture each instance of verbal behaviors. Behaviors were first coded according to the 35 CAMPIS codes and later grouped according to the CAMPIS-R categories outlined above. The two coders coded the first five transcripts from the present study together for familiarization with the unique challenges and considerations for venipuncture and this dataset. Next, separate coding of another five transcripts was undertaken, which achieved a percent agreement of 95.08%. All transcriptions were coded by the primary coder, and a random selection of 20% of the transcripts were coded by a secondary coder (percent agreement and kappa for double coded 20%: parent behavior codes = 92.41% and 0.90, respectively; child behavior codes = 95.51% and 0.93, respectively). All disagreements were resolved through discussion between the coders.

Rates of behaviors were calculated for the CAMPIS-R categories by dividing the frequency of the target code by the duration of the venipuncture in minutes (e.g., rate of parent distress-promoting behaviors = raw number of parent distress-promoting behaviors/total duration of the venipuncture in minutes). Child and adult neutral codes were not examined.

#### 2.3.3. Pain Catastrophizing Scale for Parents State (PCS-P-State)

Parents completed the PCS-P-State [29] before the venipuncture to assess their catastrophizing thoughts while anticipating their children’s upcoming procedural pain (e.g., “At this moment, to what extent do you keep thinking about how much pain your child might experience during the venipuncture?”). Using an 11-point numerical rating scale, six items are rated from 0 (not at all) to 10 (very much), with a total score ranging from 0 to 60. Higher scores indicate higher levels of catastrophizing. Construct validity and internal consistency have been demonstrated [29]. In our sample, this measure showed strong internal consistency (α = 0.90).

#### 2.3.4. Short Form of the State-Trait Anxiety Inventory-State (SF-STAI-State)

The SF-STAI-State [30] was completed by parents prior to the venipuncture to capture parent state anxiety (e.g., “I am worried”). Six items are rated on a 4-point Likert-type scale ranging from 1 (not at all) to 4 (very much), and are summed and transformed (multiplied by 20, divided by 6); this enables comparisons with the 20-item STAI-State. Total scores can vary from 20 to 80 and higher scores represent higher levels of state anxiety. The psychometric properties of the SF-STAI-State are similar to the entire STAI, including concurrent validity and internal consistency [30]. The data from our sample demonstrated acceptable internal consistency (α = 0.77).

#### 2.3.5. Electrocardiogram (ECG)

Parent cardiac activity was monitored throughout the procedure to later analyze the HRV data. The data were collected from an ECG using a BIOPAC^TM^ MP150 unit as well as a wireless BioNomadix ECG amplifier (Biopac Systems Canada Inc., Montreal, Canada) which was set to acquire data at 1000 samples per second. A standard Lead II inverted triangle configuration was used, in which an electrode was placed below each collarbone and one ground was placed below the left rib. Interbeat intervals within the ECG recording were captured with AcqKnowledge 4.2 software (Biopac Systems Canada Inc., Montreal, Canada), which was subsequently imported into Kubios HRV specialized analysis software (Premium; version 3.4.3; Kubios Ltd., Kuopio, Finland). Baseline HRV was analyzed as per existing HRV guidelines [31] and the Kubios HRV User’s Guide [32].

The raw physiological data were checked for artifacts or R-waves that had been misidentified. An automatic artifact correction algorithm, using cubic spline interpolation, was used to correct physiological artifacts [32]. Technical artifacts were fixed by editing, including cutting segments of data, and correcting mis-identified R-waves. Data were included on an epoch-by-epoch basis. The percentage of artifact editing did not surpass 10%. Missing baseline HRV values occurred as a result of technical artifacts and equipment failure during recording (see Table S1). Fifty-one parents had available HRV data at baseline.

Consistent with recommendations for short-term HRV recordings, baseline HRV was captured using a time-domain method which is less affected by respiration rate [25]. Baseline HRV was quantified by the root mean square of successive differences (RMSSD) between interbeat intervals. Baseline HRV values are provided in milliseconds, which were squared (ms^2^) and natural-log (ln) transformed to account for skewed values [33]. Parent baseline HRV was computed by extracting data during the first 30 s of the audio task (see Table S2 for the script during the first 30 s of the audio; see [24] for full scripts).

### 2.4. Data Preparation

The dataset was inspected for missing data. See Table S1 for missing cardiac and observational data. Forty-five parents had complete cardiac and observational data. Participants who had missing data on a given variable had their data removed from the corresponding analysis only; therefore, degrees of freedom range across analyses as a result of missing data and the sample size is reported for each analysis.

### 2.5. Analytic Plan

#### 2.5.1. RCT Group Variable

Before including RCT group assignment as a control variable in analyses, we sought to understand whether there were differences according to group in parent HRV, and parent and child behaviors using independent samples t-tests and Mann–Whitney U tests. There were no group differences for parent distress-promoting behavior (U = 334, *z* = −0.46, *p* = 0.65), coping-promoting behavior (U = 351, *z* = −0.16, *p* = 0.88), child distress behavior (U = 330, *z* = −0.52, *p* = 0.61), and coping behavior (*M*_difference_ = −0.25, *t*(52) = −0.47, *p* = 0.64). Group differences were found for parent HRV at baseline (*M*_difference_ = −0.58, *t*(49) = −2.05, *p* = 0.05). Post-hoc exploratory independent samples t-test with ECG-derived respiration demonstrated no group differences in respiration rate (*M*_difference_ = −0.01, *t*(49) = −0.53, *p* = 0.60), suggesting that differences in baseline HRV were not due to group differences in respiration. The pattern of findings in analyses did not vary when RCT group was entered as a control variable, thus RCT group was not controlled for. Independent samples *t*-tests were not completed with anxiety and catastrophizing as these self-report measures were obtained before pairs listened to the audio recording part of the RCT.

#### 2.5.2. Assumptions and Covariates

Data were inspected for violations of parametric assumptions. Normality was examined for all continuous variables using *z*_skewness_ (skewness/SE) and *z*_kurtosis_ (kurtosis/SE); *z* score ≥ 1.96 were considered significantly skewed or kurtotic at *p* < 0.05 [34]. The assumptions of normally distributed residuals, homoscedasticity, independent errors, and collinearity were assessed for the regression statistics [34]. The PCS-P-State (*z*_Skewness_ = 3.74, *z*_Kurtosis_ = 2.16), distress-promoting behavior (*z*_Skewness_ = 3.91), coping-promoting behavior (*z*_Skewness_ = 3.72), and distress behavior (*z*_Skewness_ = 7.87) exceeded the recommended *z*-score cutoff [34]. For the PCS-P-State, one outlier was removed as they were also missing baseline HRV, which led to a normal distribution. Outliers exceeding 3 *SD*s from the mean were detected for parent distress-promoting (*n* = 1), coping-promoting (*n* = 1), and child distress behavior (*n* = 1). These outliers were substituted using winsorizing [34]. External factors that can influence baseline HRV were examined, including age, gender, chronic pain condition, caffeine and nicotine intake, cardioactive medication use, and chronic heart or respiratory conditions [35]. Duration of the procedure was also examined as a potential control variable. No associations were found with parent age, gender, chronic pain condition, caffeine consumption, nicotine intake, or heart/respiratory conditions. However, parents who were taking cardioactive medications had lower baseline HRV (r = −0.34 *p* < 0.05). The pattern of results changed when controlling for medication use; as such, medication use was controlled for in analyses involving baseline HRV. No associations were found between the duration of the procedure and parent or child rates of behavior. Effect sizes for correlations were interpreted using Cohen’s guidelines [36].

#### 2.5.3. Main Analyses

The first objective of the study was to determine whether emotion regulation capacity (via baseline HRV) moderates the association between parents’ cognitive-affective states measured before the procedure and their behaviors during the procedure; see Figure S1. Four moderation models were performed to explore the effects of baseline HRV on the association between parent states (anxiety, catastrophizing) and behavior (distress-promoting, coping-promoting). The second goal aimed to explore if parent emotion regulation capacity (via baseline HRV) moderates the association between child distress behavior and parent behaviors (see Figure S2). Two moderation models were performed to examine parent baseline HRV, child distress behavior, and their interaction term as predictors of parent behaviors (distress- and coping-promoting). For the main analyses, child coping and distress behaviors were entered as control variables when relevant (i.e., when child coping and distress behaviors related to parent behaviors) to minimize the number of predictors in the model and preserve statistical power [36].

Sensitivity power analyses, which were completed because data collection was stopped in March of 2020 because of the COVID-19 pandemic, calculated with G*Power demonstrated that the sample of 45 parent–child pairs was powered to detect a large effect size (effect size *f*^2^ = 0.27) at power 0.80 and alpha 0.05 for moderation with three predictors [37]. The PROCESS Macro was used for moderation; to reduce multicollinearity, the predictor and moderator variables were first mean centered [38]. Interaction effects significant at *p ≤* 0.05 level were probed using simple slopes analyses, in which the association between parent states and behaviors were examined within high (i.e., +1 *SD*) and low (−1 *SD*) groups of baseline HRV.

## 3. Results

### 3.1. Participants

Sixty-one pairs participated (see Table S3 for full demographic characteristics). Children (*M_age_* = 9.95, *SD* = 1.59) were predominantly White/European (75.4%), with a chronic illness or medical condition (62.3%), with slightly more boys (54.1%) participating. Parents (*M_age_* = 42.08, *SD* = 5.77) consisted primarily of White/European (73.8%), mothers (78.7%) who were married (82%). A portion of parents had a chronic pain (29.5%) or heart/respiratory (9.8%) condition.

### 3.2. Descriptive Statistics

Table 1 presents means, standard deviations, scale range, number of cases with complete data, and correlations for study variables for descriptive purposes. Parent state catastrophizing correlated positively with rates of child distress and parent distress-promoting behavior, whereas parent state anxiety did not significantly correlate with parent or child behaviors. Parent baseline HRV correlated negatively with rates of parent coping-promoting and distress-promoting behaviors. Positive correlations were observed between parent coping-promoting and child coping behavior, and between parent distress-promoting and child distress behavior. As such, child coping was controlled for in analyses involving parent coping-promoting behaviors, and child distress was controlled for in analyses involving parent distress-promoting behaviors.

### 3.3. Moderation Analyses

**Figure 1:**
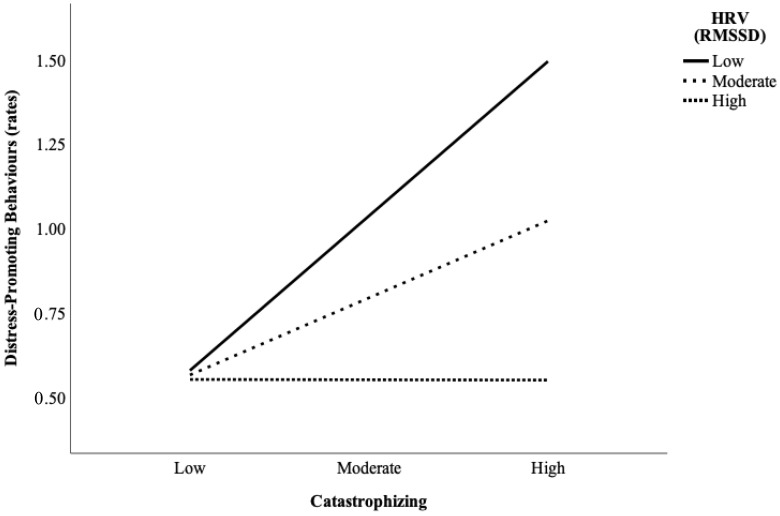
Parent baseline HRV as a moderator between parental catastrophizing about child pain and distress-promoting behaviors.

The first objective of this study was to examine whether parent baseline HRV moderated the association between parent states (anxiety, catastrophizing) and parent behaviors (coping-promoting, distress-promoting), while controlling for concurrent child behaviors. The first moderation model examined parent catastrophizing, baseline HRV, and catastrophizing by HRV interaction as predictors (although parent and child behaviors were measured concurrently, the term predictor will be used when discussing the moderation analyses to remain consistent with regression terminology) of parent coping-promoting behaviors. Child coping and parent medication use were entered as control variables. Results indicated there was no significant main effect of parent state catastrophizing, parent baseline HRV, or catastrophizing by HRV interaction effect (see Table 2). The second moderation model examined parent catastrophizing, baseline HRV, and catastrophizing by HRV interaction as predictors of parent distress-promoting behaviors. Child distress and parent medication use were entered as control variables. Results suggest a significant negative effect of parent baseline HRV and a positive main effect of parent state catastrophizing (see Table 2). A significant catastrophizing by HRV interaction effect emerged. Results from the simple slopes analysis show that the positive relation between catastrophizing and distress-promoting behaviors was strongest among individuals with low (*t* = 2.79, *p* = 0.01) and moderate levels of parent baseline HRV (*t* = 2.18, *p* = 0.04; see Figure 1). The pattern of findings remained after controlling for parent anxiety, which was not included in the model to preserve statistical power.

The third moderation model examined parent anxiety, baseline HRV, and anxiety x HRV interaction as predictors of parent coping-promoting behaviors. Child coping and parent medication use were entered as control variables. Results indicated no significant main effect or interaction effects (see Table 2). The fourth moderation model examined parent anxiety, baseline HRV, and anxiety × HRV interaction as predictors of parent distress-promoting behaviors. Child distress and parent medication use were entered as a control variable. Results indicated no significant main effect or interaction effects (see Table 2).

The second objective of the study was to determine whether parent emotion regulation capacity (via baseline HRV) affects the extent to which children’s displays of distress relate to parent coping-promoting and distress-promoting behavior. In the first moderation model, parent baseline HRV, child distress behavior, and their interaction term were entered as predictors of parent coping-promoting behavior. Child coping behavior and parent medication use were entered as control variables. There was a main effect of parent baseline HRV, and no main effect of child distress or interaction effect in predicting coping-promoting behaviors. In the second moderation model, parent baseline HRV, child distress behavior, and their interaction term were entered as predictors of parent distress-promoting behavior. Parent medication use was entered as a control variable. There was a significant main effect of child distress behavior and parent baseline HRV. There was no interaction effect in predicting parent distress-promoting behaviors (see Table 3).

## 4. Discussion

The affective-motivational model (AMM) of interpersonal pain dynamics [1] posits emotion regulation is central in influencing the way parents respond to their child’s pain; however, empirical evidence is scant on why parents act the way they do during their children’s medical procedures. We examined whether parent baseline HRV, as an index of emotion regulation capacity, could play a role. We first looked at whether parent emotion regulation capacity shapes the way in which parents’ cognitive-affective states relate to their behaviors. Consistent with expectations, parent baseline HRV moderated the association between parent state catastrophizing and distress-promoting behaviors; parents experiencing high catastrophic thoughts about their child’s pain had the highest rates of distress-promoting behaviors if their baseline HRV was low. Catastrophizing, characterized by a focus on and magnification of the threat value of painful stimuli, commonly relates positively to distress-promoting behaviors (e.g., [11]); however, we show that this association varies based on parent baseline HRV. Parents with low baseline HRV, or low capacity to regulate their emotions, appear to be most vulnerable to engaging in unhelpful verbal behaviors during their child’s venipuncture, particularly if they were experiencing catastrophic thoughts before the procedure, which aligns with lab-based findings [17]. We extend these findings to a clinical context, controlling for child distress behaviors. Our results are similar to findings by Vervoort and colleagues [19] demonstrating that parental HRV moderated the extent to which children’s facial displays of pain related to pain control behaviors; parents with low HRV engaged in greater pain control behaviors during a lab-based pain task.

Contrary to expectations, baseline HRV did not moderate the associations between parent state anxiety and parent behaviors, or between parent state catastrophizing and coping-promoting behaviors. These findings may be partly understood by examining the operationalization of our variables and should be considered in the broader context of the pediatric pain literature. Generally, our measures correlated as expected based on existing work (e.g., [12]); parents with higher catastrophic thinking engaged in greater distress-promoting behavior and had children with higher distress. There were no correlations between parent state anxiety and parent distress-promoting and child distress behaviors, which is consistent with literature on immunizations and oncology procedures [13,39]. This may be partly explained by the measure’s specificity to the pain stimulus; parent catastrophizing was assessed by prompting parents to think about their child’s upcoming procedure, whereas state anxiety was measured by having parents indicate how tense, upset, and worried they felt in the moment. Future work would benefit from asking parents directly about their emotions (anxiety, fear, distress) in relation to their child undergoing venipuncture. However, our effect size (*r* = 0.22) is similar to the small effect observed between state anxiety and parent behaviors in other work (*r* = 0.24, *p* < 0.10; e.g., [18]), which speaks to the need to replicate this study with a larger sample. Taken together, our preliminary findings suggest that parental emotion regulation capacity may contribute the most to parent distress-promoting behaviors, particularly when parents are experiencing unhelpful thoughts about their child’s pain.

The AMM highlights expressions of pain in shaping the responses of observers. Thus, a second objective was to determine whether parents’ emotion regulation capacity shapes the degree to which children’s displays of distress relate to parent behaviors. Consistent with previous literature, child distress behavior was a strong correlate of parent distress-promoting behavior [5]. This finding reinforces the importance of the social context in shaping both parent and child verbal behaviors during painful procedures. Parental verbalizations serve several communicative purposes, such as providing signs of their emotional states and guiding their child’s attention; in the context of a venipuncture, parents verbalizations may cue the child to use a coping tool (e.g., “take a deep breath”) or focus their attention on the procedure (e.g., “I know you don’t want to do this, but it will be over soon”). Communication between parent and child is bidirectional, with child verbalizations similarly communicating their emotional states and the extent to which they are coping [40]. However, even after controlling for the effects of child behaviors, parent baseline HRV related negatively to both parent distress- and coping-promoting behaviors. Thus, parents who had greater regulation capacity before the procedure engaged in less frequent coping- and distress-promoting behaviors which may suggest these parents felt less threatened by the procedure and therefore less need to engage their child verbally. This aligns with lab-based findings, reporting an inverse association between parent HRV during child pain and rate of coping-promoting behaviors [17]. There were no moderation effects of parent baseline HRV between child distress and parent behaviors. In this way, parent baseline HRV may be particularly relevant for understanding the association between their own cognitions and subsequent behaviors. To illustrate, parents with low baseline HRV experiencing unhelpful thoughts about their child’s pain prior to the procedure may have less resources to regulate arising distress, thereby engaging in greater distress-promoting behaviors. However, parent baseline HRV may have a greater role in shaping parent responses to their child’s nonverbal behaviors (e.g., facial expressions [19]), which should be explored in future work. Notably, findings from our larger study suggest that children’s baseline HRV is relevant to understanding parent–child verbal interactions; that is, children’s baseline HRV moderated the relation between parent distress-promoting behaviors and child distress behaviors ([23]). Together, our findings suggest low baseline HRV places certain parents at greater risk of engaging in distress-promoting behaviors, and positions children to experience more distress. Sequential analyses examining the temporal association between parent and child behaviors may be used to shed light on whether low baseline HRV may place parents at greater risk of using distress-promoting behaviors prior to or following children’s distress.

### 4.1. Strengths and Limitations

This study is the first to utilize psychophysiological, self-report, and behavioral data to explore parents’ baseline HRV, cognitive-affective states, and child behaviors in relation to parent behaviors throughout pediatric venipuncture. Parent and child verbal behaviors were measured using a rigorous and intensive transcription and coding approach using the CAMPIS-R. Moreover, the observational approach allowed for spontaneous interactions, thereby enhancing the ecologically validity of the interpersonal data [41]. Following HRV guidelines (e.g., [25]), we assessed and controlled for extraneous factors that have the potential to influence baseline HRV. This comprehensive, multi-method approach contributes to our understanding of the biopsychosocial factors shaping parent’s experience during their child’s acute pain, which informs theoretical models of pain and has the potential to guide parent interventions.

Several limitations of this study warrant further discussion and present fruitful avenues for future research. Replication with a larger sample is warranted, particularly to provide power for small and moderate effects and permit more advanced statistical models (e.g., multi-level modeling). Although 30 s epochs of RMSSD have been deemed to be a valid index of HRV (e.g., [42]), future research may examine the extent to which this baseline measure relates to HRV measured for longer durations in a clinical context. Parent and child verbal behaviors were grouped based on the CAMPIS-R; examining all the individual CAMPIS codes may help to further elucidate which type of distress-promoting behaviors parents with low baseline HRV may be more likely to engage in. Parent and child verbal behaviors were studied given that the focus of this paper was to understand contributors to parent verbal behaviors; however, nonverbal behaviors such as facial expressions provide a rich source of emotive information that may further our understanding of parent responses and parent–child interactions [19,43]. Notably, more than half of our sample consisted of children with a chronic illness or medical condition, which is comparable other work studying parent–child interactions during pediatric venipuncture [44]. Given that children with health conditions tend to undergo needle procedures more often compared to children without such conditions and past experiences shape future responses, examining how the quality of children and their parents’ prior needle experiences shapes their future responses and behaviors is warranted. More broadly, measuring the typical dynamics of the parent–child relationship and their attachment style paired with HRV is expected to inform how children express and regulate their needle-related distress, and how parents appraise and respond to their child’s needle-related needs (e.g., [45]). Further, our sample largely consists of families who self-identified into groups that hold privilege in terms of ethnicity (75% White/European) and education (77% college or university level education), which may associate with better experiences with the medical system. In contrast, it is possible that a different pattern of findings might emerge with families from marginalized backgrounds given the documented disparities in health status and access to care by ethnicity and income (e.g., [46,47]). Moreover, evidence suggests that the impact of certain parental verbalizations (e.g., distraction) during children’s acute pain varies by socioeconomic status [48]. As such, including a sample with a range of sociodemographic backgrounds, who may have different experiences with the medical system, is needed. This study did not account for nurse behaviors, which is an additional social factor that should be explored in future research. A natural extension of this work is to examine parent and child behaviors based on the phase of the procedure, as verbal behaviors have been shown to differ significantly based on the procedural phase [3].Lastly, the present paper aimed to study parent’s baseline HRV to understand their emotion regulation capacity and how it may shape their verbal behaviors; however, incorporating a self-report measure of emotion regulation would inform the strategies parents employ to regulate any arising emotions.

### 4.2. Implications

Findings from this study may inform theoretical and clinical implications. We provide empirical support for the AMM [1] by illustrating the moderating role of parent emotion regulation capacity as measured by baseline HRV. In particular, our results suggest that low baseline HRV serves as a risk factor for parents to engage in a group of behaviors that are less attuned to their child’s needs, particularly in parents who view pain as highly threatening. In terms of clinical implications, consistent with arguments put forth by Caes and colleagues [12], modifying catastrophic thinking may not be required to reduce parent distress-promoting behaviors; rather, efforts may be better focused on supporting parents in how they respond to such thoughts, which may be accomplished through emotion regulation strategies and/or enhancing HRV. At a biological level, HRV may be enhanced through changes to daily routines, biofeedback training, and relaxation and mindfulness practice [49,50]. Enhancing HRV is likely to be beneficial beyond the venipuncture context given results that low HRV increases parents’ likelihood of engaging in other unhelpful pain-related behaviors (e.g., attempts to control pain [19]), in addition to findings that high HRV generally relates to increased health and wellbeing [51]. Emotion regulation skills at the psychological level may involve cognitive reappraisal or acceptance [52], the latter of which may be particularly beneficial in this context. Individuals with elevated catastrophizing are especially attuned to pain-related signals and struggle to shift attention away from painful stimuli [18]; thus, strategies that alter the ways in which parents relate to their child’s pain may be particularly helpful [24].

## 5. Conclusions

Taken together, our findings echo the notion that the pediatric venipuncture experience is embedded within a social context in which parent and child behaviors are strongly influenced by one another. Psychologically, the extent to which parent cognitive appraisals about their child’s pain relate to their distress-promoting behaviors depends on parent emotion regulation capacity. Biologically, low levels of HRV may place certain parents at greater risk of using behaviors that are less attuned with their child’s needs.

## Figures and Tables

**Table 1:** Descriptive statistics and correlations between study variables with confidence intervals (*n* = 45 to 60).

Variable	*M (SD)*	Range	*n*	1	2	3	4	5	6
1. Catastrophizing	14.08 (11.73)	0–44	60						
2. Anxiety	34.32 (11.76)	20–60	61	0.53 ** [0.32, 0.69]					
3. Parent baseline HRV ^a^	7.00 (1.05)	4.81–8.98	51	−0.15 [−0.41, 0.13]	−0.24 [−0.48, 0.03]				
4. Child coping behavior	3.09 (1.91)	0–8.39	54	−0.14 [−0.40, 0.14]	−0.23 [−0.46, 0.05]	−0.17 [−0.45, 0.14]			
5. Child distress behavior ^b^	1.46 (1.74)	0–10.00	54	0.31 * [0.04, 0.53]	0.08 [−0.19, 0.34]	−0.11 [−0.40, 0.19]	0.24 [−0.02, 0.47]		
6. Coping-promoting behavior ^b^	3.33 (2.01)	0.57–9.45	54	0.05 [−0.22, 0.32]	−0.16 [−0.41, 0.12]	−0.38 * [−0.61, −0.09]	0.62 ** [0.40, 0.78]	−0.06 [−0.35, 0.23]	
7. Distress-promoting behavior ^b^	0.87 (0.92)	0–3.83	54	0.35 * [0.08, 0.57]	0.22 [−0.05, 0.46]	−0.38 * [−0.61, −0.09]	0.16 [−0.14, 0.43]	0.52 ** [0.27, 0.71]	0.18 [−0.11, 0.50]

Note. *M* = mean; *SD* = standard deviation; range = observed range; *n* = number of valid cases. Catastrophizing = parent state catastrophizing as assessed by the PCS-P-State; anxiety = parent state anxiety as assessed by the SF-STAI-State; baseline HRV = heart rate variability quantified by the root mean square of successive differences (RMSSD; log transformed, reported in ms^2^) during the first 30 s of an audio task. Behaviors are reported in rates (frequency of target behavior/duration of the venipuncture). Values in square brackets represent the 95% confidence interval for each correlation. * indicates *p* < 0.05. ** indicates *p* < 0.01. ^a^ Partial correlations controlling for medication use. ^b^ Spearman’s rank correlation coefficient.

**Table 2:** Summary of regression analysis with parent catastrophizing and anxiety, parent baseline HRV and their interaction term as predictors of parent verbal behaviors, controlling for child behaviors and parent medication (*n* = 45).

Predictor Variable	*b (SE)*	*t*	*p*	95% Confidence Interval for *b*	
				Lower Bound	Upper Bound
*Model 1 Predictor = Parent Catastrophizing, Criterion = Parent Coping-Promoting Behavior*					
Parent catastrophizing	0.00 (0.02)	0.16	0.88	−0.04	0.04
Parent baseline HRV	−0.37 (0.23)	−1.59	0.12	−0.84	0.10
Parent catastrophizing X parent baseline HRV	−0.02 (0.02)	−0.87	0.38	−0.06	0.02
*Covariates*					
Child coping behaviors	0.64 (0.12)	5.54	<0.001	0.41	0.88
Parent medication	−0.51 (0.48)	−1.06	0.29	−1.48	0.46
*Model 2 Predictor = Parent Catastrophizing, Criterion = Parent Distress-Promoting Behavior*					
Parent catastrophizing	0.02 (0.01)	1.92	0.06	−0.00	0.03
Parent baseline HRV	−0.25 (0.10)	−2.40	0.02	−0.46	−0.04
Parent catastrophizing X parent baseline HRV	−0.02 (0.01)	−2.03	0.05 ^a^	−0.04	−0.00
*Covariates*					
Child distress behaviors	0.25 (0.06)	4.23	<0.001	0.13	0.37
Parent medication	−0.33 (0.21)	−1.52	0.14	−0.76	0.11
*Model 3 Predictor = Parent Anxiety, Criterion = Parent Coping-Promoting Behavior*					
Parent anxiety	−0.00 (0.02)	−0.05	0.96	−0.05	0.04
Parent baseline HRV	−0.37 (0.24)	−1.53	0.13	−0.86	0.12
Parent anxiety X parent baseline HRV	−0.01 (0.02)	−0.65	0.52	−0.05	0.03
*Covariates*					
Child coping behaviors	0.65 (0.12)	5.45	<0.001	0.41	0.88
Parent medication	−0.53 (0.48)	−1.11	0.27	−1.50	0.44
*Model 4 Predictor = Parent Anxiety, Criterion = Parent Distress-Promoting Behavior*					
Parent anxiety	0.02 (0.01)	1.52	0.14	−0.01	0.04
Parent baseline HRV	−0.22 (0.12)	−1.94	0.06	−0.46	0.01
Parent anxiety X parent baseline HRV	−0.01 (0.01)	−0.71	0.48	−0.03	0.01
*Covariates*					
Child distress behaviors	0.16 (0.04)	3.96	<0.001	0.08	0.24
Parent medication	−0.40 (0.23)	−1.77	0.09	−0.87	0.06

*Note*. Parent catastrophizing = parent state catastrophizing as assessed by the PCS-P-State; parent anxiety = parent state anxiety as captured by the SF-STAI-State; parent baseline HRV = heart rate variability measured by the root mean square of successive differences (RMSSD; log transformed, reported in ms^2^) during the first 30 s of an audio task; behaviors are reported in rates (frequency of target behavior/duration of the venipuncture). *R*^2^ = 0.50 for model 1; *R^2^* = 0.56 for model 2; *R*^2^ = 0.50 for model 3; *R^2^* = 0.49 for model 4. ^a^ *p* = 0.049.

**Table 3:** Summary of regression analysis with parent baseline HRV, child distress behavior, and their interaction terms as predictors of parent behavior (*n* = 45).

Predictor Variable	*b (SE)*	*t*	*p*	95% Confidence Interval for *b*	
				Lower Bound	Upper Bound
*Model 5 Criterion = Parent Coping-Promoting Behavior*					
Child distress	−0.11 (0.22)	−0.68	0.50	−0.44	0.22
Parent baseline HRV	−0.46 (0.31)	−2.06	0.05	−0.90	−0.01
Child distress X HRV	0.12 (0.14)	1.24	0.22	−0.08	0.32
*Covariates*					
Child coping behavior	0.69 (0.11)	6.32	<0.001	0.47	0.91
Parent medication	−0.35 (0.43)	−0.77	0.44	−1.26	0.56
*Model 6 Criterion = Parent Distress-Promoting Behavior*					
Child distress	0.28 (0.08)	3.46	<0.01	0.12	0.44
Parent baseline HRV	−0.29 (0.11)	−2.58	0.01	−0.51	−0.06
Child distress × HRV	−0.01 (0.05)	−0.16	0.88	−0.11	0.10
*Covariates*					
Parent medication	−0.38 (0.23)	−1.65	0.11	−0.84	0.08

*Note*. Parent baseline HRV = heart rate variability quantified by the root mean square of successive differences (RMSSD; log transformed, reported in ms^2^) during the first 30 s of an audio task; behaviors are reported in rates (frequency of target behavior/duration of the venipuncture). *R*^2^ = 0.55 for model 5; *R*^2^ = 0.48 for model 6.

## Data Availability

Research data are not shared due to privacy or ethical restrictions.

## Supplementary Materials

The following supporting information can be downloaded at: https://www.mdpi.com/article/10.3390/children9071000/s1, Figure S1: Visual depiction of objective 1, examining whether emotion regulation capacity (via baseline HRV) moderates the association between parents’ cognitive-affective states (measured before the procedure) and their behaviors (during the entire procedure); Figure S2: Visual depiction of objective 2, examining whether parent emotion regulation capacity (via baseline HRV) moderates the association between child displays of distress and parent behaviors; Table S1: Reasons for cardiac and observational data not being usable from a sample of 61 parents; Table S2: Excerpts from scripts for audio recording during baseline HRV based on participant and RCT group. For full scripts see Moline et al. [24]; Table S3: Demographic characteristics of the sample (*n* = 61).
