# Supplementary material for: Biopsychosocial Contributors to Parent Behaviors during Child Venipuncture
Source: Children (Basel). 2022 Jul 2;9(7):1000. doi: 10.3390/children9071000 (PMC9318291; doi:10.3390/children9071000)
Supplement: Supplementary file 1 [file children-09-01000-s001.zip › children-1778687-supplementary.pdf]

Table S1. Reasons for cardiac and observational data not being usable from a sample of 61 parents

Cardiac data		Observational data	
Equipment failure	4	Missing audio	4
Technical artifacts	6	Speaking another language/ unintelligible transcripts	3
Number retained:	51	Number retained:	54
Number of participants with complete cardiac and observational data:		45	

Table S2. Excerpts from scripts for audio recording during baseline HRV based on RCT group. For full scripts see Moline et al. [24]

	Baseline
Parent	
Mindfulness group	*During the next few minutes, you will be guided through an activity that you can use before and during your child's needle. When you're ready, you can get your body into a comfortable yet alert position. Close your eyes and take a slow, deep breath…in through your nose.. 1,2,3,4,5*
Control group	*During the next few minutes, you will be guided through an activity that you will learn and practice before the needle and use during the needle …When you're ready, you can start by allowing your mind to roam; there is no need to focus on anything in particular… Just let your mind wander…*

Table S3. Demographic characteristics of the sample (n = 61)

Variable	Frequency (%)
**Child Characteristics**	
Gender	
Female	28 (45.9)
Male	33 (54.1)
Age ($M_{years} \pm SD$)	9.95 (1.59)
Ethnicity	
Indigenous/First Nation/Metis	0 (0)
White/European	46 (75.4)
Black/African/Caribbean	5 (8.2)
Southeast Asian (e.g., Chinese, Japanese, Korean, etc.)	3 (4.9)
Arab (Saudi Arabian, Palestinian, Iraqi. etc.)	5 (8.2)
South Asian (East Indian, Sri Lankan, etc.)	2 (3.3)
Latin American (Costa Rican, Guatemalan, Brazilian, etc.)	2 (3.3)
West Asian (Iranian, Afghani)	1 (1.6)
Other	3 (4.9)
Chronic illness or medical condition	
Yes	38 (62.3)
No	23 (37.7)
Cardioactive medication use	

Yes	34 (55.7)
No	27 (44.3)
**Parent Characteristics**	
Relationship to child	
Mother	47 (78.7)
Father	12 (19.7)
Other	1 (1.6)
Age ($M_{\text{years}} \pm SD$)	42.08 (5.77)
Gender	
Female	49 (80.3)
Male	12 (19.7)
Ethnicity	
Indigenous/First Nation/Metis	0 (0)
White/European	45 (73.8)
Black/African/Caribbean	4 (6.6)
Southeast Asian (e.g., Chinese, Japanese, Korean, etc.)	1 (1.6)
Arab (Saudi Arabian, Palestinian, Iraqi. etc.)	5 (8.2)
South Asian (East Indian, Sri Lankan, etc.)	2 (3.3)
Latin American (Costa Rican, Guatemalan, Brazilian, etc.)	2 (3.3)
West Asian (Iranian, Afghani)	1 (1.6)
Other	2 (3.3)
Marital status	
Married	50 (82)
Common Law	4 (6.6)
Divorced/Separated	7 (11.5)
Highest level of education	
Completed high school	2 (3.3)
Some college/university	11 (18)
Apprenticeship training and trades	1 (1.6)
Completed college/university	34 (55.7)
Some graduate education	1 (1.6)
Completed graduate education	8 (13.1)
Professional degree	4 (6.6)
Chronic pain condition	
Yes	18 (29.5)
No	43 (70.5)
Chronic heart/respiratory condition	
Yes	6 (9.8)
No	55 (90.2)
Caffeine consumption in the past 2 hours	
Yes	32 (52.5)
No	29 (47.5)
Nicotine consumption in the past 2 hours	
Yes	6 (9.8)
No	55 (90.2)
Cardioactive medication use	
Yes	26 (42.6)

No	35 (57.4)

Note. Parents reported on child demographic information. Ethnicity may exceed 61 given that participants could select more than one option.

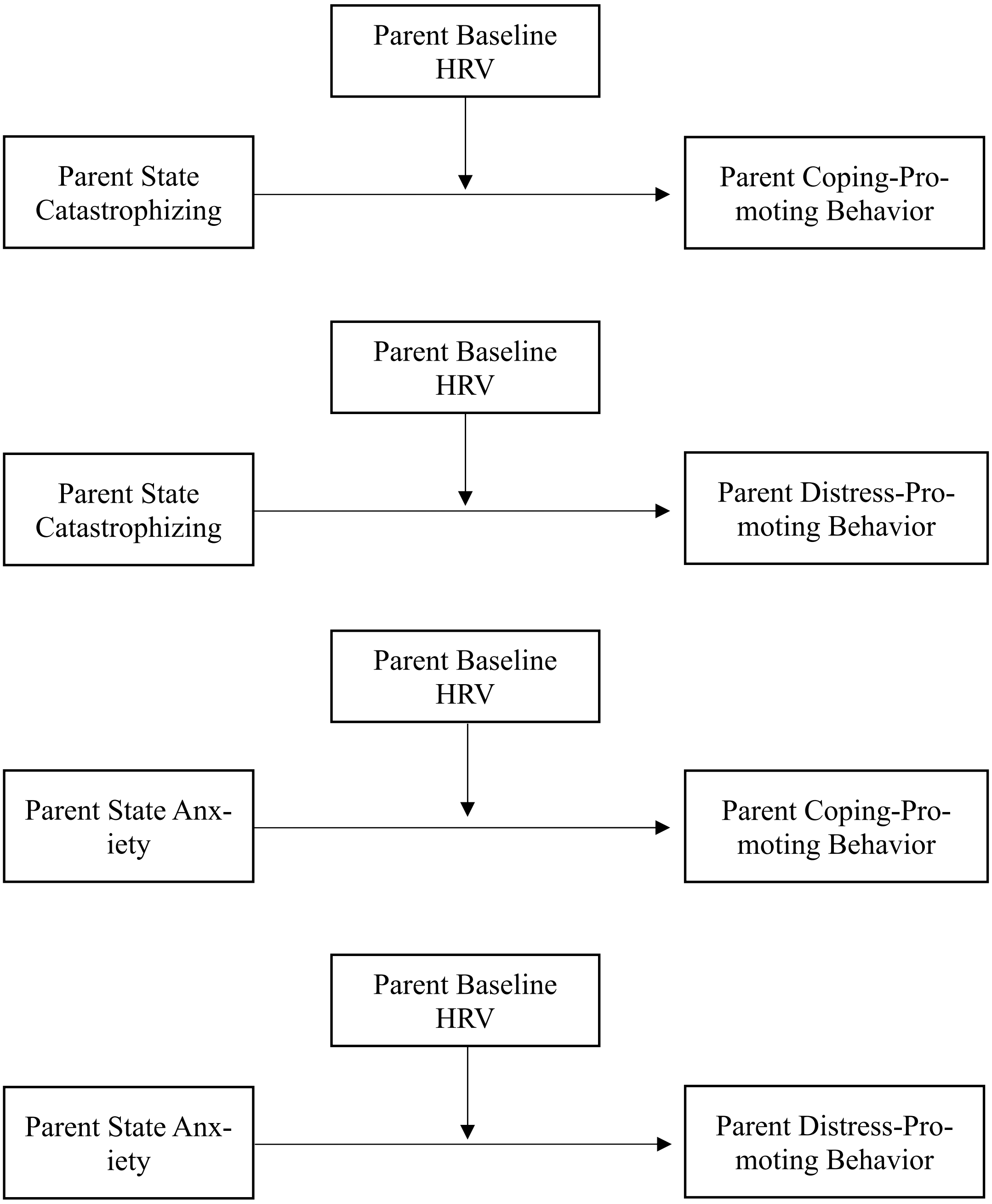

Figure S1. Visual depiction of objective 1, examining whether emotion regulation capacity (via baseline HRV) moderates the association between parents' cognitive-affective states (measured before the procedure) and their behaviors (during the entire procedure).

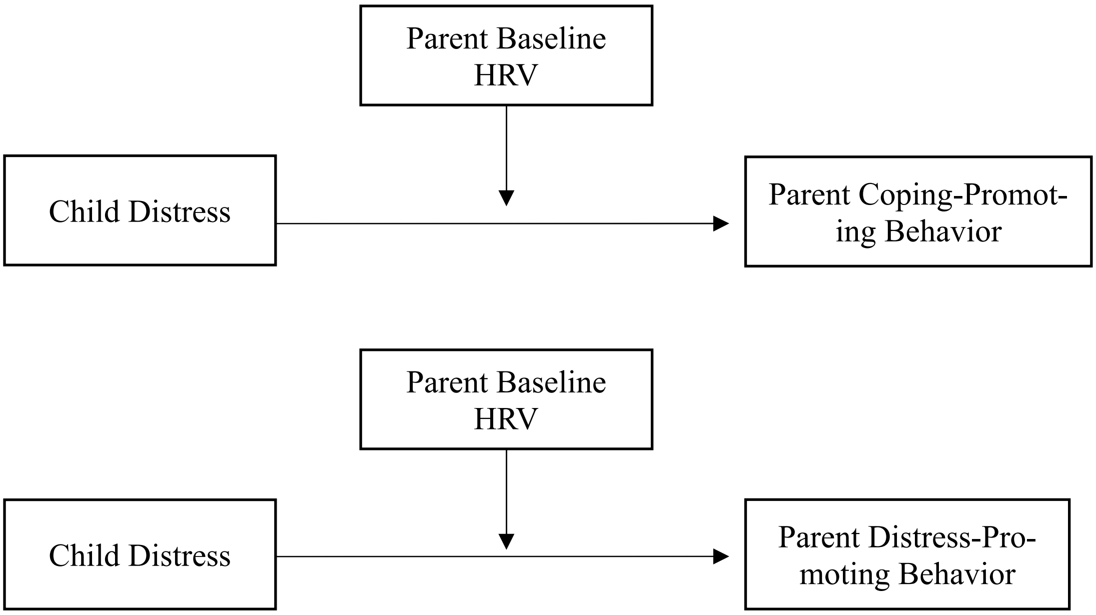

Figure S2. Visual depiction of objective 2, examining whether parent emotion regulation capacity (via baseline HRV) moderates the association between child displays of distress and parent behaviors.
